# Clinical Vulnerability Refines Rhythm‐Related Outcomes in Older Patients Undergoing Pulmonary Vein Isolation for Atrial Fibrillation: An Observational Cohort Study

**DOI:** 10.1002/joa3.70461

**Published:** 2026-09-25

**Authors:** Yazan Mohsen, Jascha Fernholz, Lucas Steffens, Iryna Novikov, Shreeram Sabareesan, Ishan Vatsaraj, Kensuke Sakata, Moritz Knitter, Henning Horlitz, Marc Horlitz, Mustafa Zakkar, Riyaz Somani, G. Andre Ng, Dennis Rottländer, Florian Stöckigt, Ibrahim Antoun

**Affiliations:** ^1^ Department of Biomedical Engineering Johns Hopkins University Baltimore Maryland USA; ^2^ Department of Cardiology, Electrophysiology and Rhythmology Krankenhaus Porz am Rhein Cologne Germany; ^3^ Department of Cardiology, School of Medicine University Witten/Herdecke Witten Germany; ^4^ Maastricht University Maastricht the Netherlands; ^5^ Division of Cardiovascular Sciences, College of Life Sciences University of Leicester, Glenfield Hospital Leicester Leicestershire UK; ^6^ Department of Cardiology University Hospitals of Leicester NHS Trust, Glenfield Hospital Leicester Leicestershire UK; ^7^ Department of Internal Medicine II University Hospital Bonn Bonn Germany

**Keywords:** atrial fibrillation, cardioversion, catheter ablation, clinical vulnerability, elderly, pulmonary vein isolation, recurrence, repeat ablation

## Abstract

**Introduction:**

Chronological age is often considered when selecting patients for atrial fibrillation (AF) ablation, but age alone may not identify which older patients are most likely to require recurrent rhythm‐related care after pulmonary vein isolation (PVI).

**Methods:**

We conducted a single‐center observational study of consecutive patients undergoing PVI. The principal registry cohort assessed the same‐center recurrence‐related rhythm‐intervention endpoint, defined as the first post‐index repeat PVI or electrical cardioversion. A nested active 2022 cohort assessed broader actively ascertained recurrence. Age was analyzed as < 50, 50–74, and ≥ 75. An exploratory elderly vulnerability score assigned one point each for documented persistent AF, heart failure, chronic kidney disease, and valve disease/surgery.

**Results:**

The registry cohort included 2559 patients and 580 same‐center rhythm‐intervention events. Event rates increased across age groups: 14.2% in patients aged < 50, 20.8% in those aged 50–74, and 29.8% in those aged ≥ 75. Age ≥ 75 was independently associated with higher risk versus age 50–74 in adjusted registry Cox analysis, HR: 1.35, 95% CI: 1.12–1.64, *p* = 0.002, after 90‐day blanking, HR: 1.37, 95% CI: 1.12–1.69, *p* = 0.002, and in the active cohort, HR: 1.46, 95% CI: 1.01–2.11, *p* = 0.042. Elderly Score 0 patients were not significantly different from patients aged 50–74 in registry analysis, aHR: 1.13, 95% CI: 0.79–1.63, *p* = 0.5, or active analysis, aHR: 1.01, 95% CI: 0.49–2.09, *p* = 0.975, whereas Score ≥ 2 identified higher event burden in both the registry cohort, aHR: 1.85, 95% CI: 1.45–2.35, *p* < 0.001, and active cohort, aHR: 1.95, 95% CI: 1.23–3.12, *p* = 0.005.

**Conclusion:**

Age ≥ 75 was associated with a higher rate of same‐center recurrence‐related rhythm interventions after PVI, but the risk varied substantially. Clinical vulnerability, not chronological age alone, may better inform counseling and selection of older patients.

## Graphical abstract

In patients undergoing pulmonary vein isolation, age ≥ 75 years was associated with greater recurrence‐related rhythm intervention. However, outcomes varied markedly among older adults. A simple score incorporating persistent AF, heart failure, chronic kidney disease, and valve disease identified elderly patients with low or high clinical vulnerability.

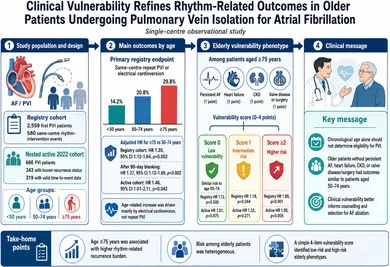

## 1 Introduction

Catheter ablation is an established rhythm‐control strategy for atrial fibrillation (AF), and pulmonary vein isolation (PVI) remains the procedural cornerstone of AF ablation [1]. Contemporary guidelines support catheter ablation to reduce AF symptoms and recurrence in appropriately selected patients, with increasing emphasis on shared decision‐making and management of modifiable clinical risk factors.

Older adults now represent a growing proportion of patients considered for AF ablation [2]. This reflects population aging, higher AF prevalence with age, improved procedural safety, and the clinical burden imposed by recurrent symptomatic AF. However, the expected benefit of ablation in elderly patients remains difficult to estimate at the bedside. Older patients are more likely to have persistent AF, atrial substrate progression, renal dysfunction, heart failure, vascular disease, and valvular disease, all of which may influence rhythm outcomes and post‐ablation management [3].

Previous studies and meta‐analyses suggest that AF ablation in patients aged 75 years or older can be clinically useful, but recurrence and complication rates are generally higher than in younger patients [4]. These data support caution but do not establish chronological age as a sufficient selection tool. Some elderly patients have limited comorbidity, paroxysmal AF, preserved renal function, and no major structural valve disease. Others have advanced substrate and competing clinical vulnerability. Treating these groups as a single age‐defined category may obscure clinically important heterogeneity.

We therefore evaluated age, clinical vulnerability, and recurrence‐related outcomes after AF ablation with PVI in a large single‐center registry and a nested actively followed cohort. We aimed to identify an interpretable age partition, assess whether age ≥ 75 years was independently associated with recurrence‐related interventions, explore subgroup patterns by sex and AF phenotype, and test whether a simple exploratory elderly vulnerability phenotype could separate older patients with favorable versus unfavorable post‐ablation outcomes.

## 2 Methods

### 2.1 Study Design and Setting

We conducted a single‐center observational study of consecutive patients undergoing catheter ablation for AF with PVI. The analysis used two overlapping populations with different ascertainment of recurrence. The principal registry cohort evaluated same‐center recurrence‐related interventions after PVI ablation. The nested active 2022 cohort was analyzed separately because it used more intensive follow‐up and a broader actively ascertained recurrence endpoint. The active cohort was not treated as external validation because it was nested within the institutional registry framework. The study adhered to the STROBE reporting guidelines [5].

### 2.2 Participants

The principal registry cohort included the first eligible PVI ablation per patient. Eligibility required AF as the target arrhythmia and procedural documentation consistent with PVI. When more than one eligible PVI procedure was present for a patient, the first eligible ablation during the study period was retained as the index procedure. Subsequent repeat PVI procedures and electrical cardioversions were retained as potential outcome events. The final registry cohort contained 2559 patients and 580 primary same‐center events.

The nested active 2022 cohort included 666 patients who underwent PVI ablation in 2022. Of these, 343 had known active recurrence status, and 319 contributed valid active time‐to‐event data, including 165 timed recurrence events.

### 2.3 Clinical Data and Definitions

Baseline data were abstracted from clinical documentation, procedure reports, discharge summaries, diagnosis lists, medication lists, electrocardiograms, laboratory results, and echocardiographic information when available. Variables included age, sex, body mass index (BMI), AF phenotype, hypertension, diabetes mellitus, heart failure, coronary artery disease or coronary revascularization, prior stroke or transient ischemic attack, chronic kidney disease (CKD), obstructive sleep apnea, tachycardiomyopathy or cardiomyopathy coding, peripheral vascular disease, valvular heart disease or valve surgery, thyroid disease, smoking history, medication classes, and peri‐procedural laboratory values. Comorbidities were treated as clinically documented diagnoses rather than administrative claims. Persistent AF was defined according to the AF phenotype documented by the treating clinical team in the source record. In the registry cohort, absence of documented persistent AF was not assumed to represent confirmed paroxysmal AF when AF phenotype documentation was incomplete. Heart failure was defined as a clinically documented diagnosis in the source records.

CKD was defined as KDIGO G3a or worse, based on clinically documented CKD in the source records and/or baseline eGFR < 60 mL/min/1.73 m^2^ when available. Valve disease/surgery was defined as any coded mitral insufficiency, tricuspid insufficiency, aortic insufficiency, aortic stenosis, or prior valve surgery. Severity and valve subtype were not used as separate primary score components.

### 2.4 Age Exposure

Age was the primary exposure. Candidate age partitions were explored within clinically interpretable ranges before selecting the main categories. Young thresholds of < 40, < 50, < 55, < 60, and < 65 years were combined with older thresholds of ≥ 75 years; an alternative ≥ 80 years threshold was also assessed. The final main age grouping was < 50, 50–74, and ≥ 75 years. This partition preserved an early‐onset younger AF ablation group, retained adequate event counts, and provided Kaplan–Meier separation in both the registry and active 2022 cohorts. Because cutoff selection was data‐guided, this analysis should be interpreted as exploratory and internally derived.

### 2.5 Ablation Procedures

All ablation procedures were performed in an experienced electrophysiology laboratory in accordance with contemporary clinical practice. PVI was the procedural endpoint for AF ablation. Ablation modality, including cryoballoon PVI or radiofrequency/3D‐mapping‐guided PVI, and additional lesion sets were chosen by the treating electrophysiologist according to AF phenotype, prior ablation history, concomitant atrial flutter, substrate, and intra‐procedural findings. Registry procedural variables were derived from procedure‐text indicators. The active 2022 dataset also contained detailed procedural variables.

Procedural safety was assessed in the nested active 2022 cohort, where structured complication fields were available. Complications included vascular complications, AV fistula, pseudoaneurysm/aneurysm, groin operation, transfusion, air embolism, phrenic nerve lesion, CPR, stroke/TIA, pericardial effusion/tamponade, and procedure‐related death. Structured registry‐wide procedural complication data were not available.

### 2.6 Endpoints

All patients were scheduled for 7‐day Holter monitoring approximately 90 days after ablation, followed by clinical review either in person or by telephone. Recurrence documented during this standardized post‐blanking Holter assessment or follow‐up consultation was recorded when available. Longitudinal outcome ascertainment then differed by cohort.

In the principal registry cohort, the primary endpoint was the same‐center rhythm‐intervention endpoint, defined as the first post‐index repeat PVI or electrical cardioversion performed at the study center. Electrical cardioversion was included because, after PVI, it represents clinically recognized recurrent atrial arrhythmia requiring rhythm‐control intervention. This definition was applied identically across all age groups. This endpoint was selected because these events could be verified with high certainty in the retrospective registry and reflect clinically actionable rhythm‐related care after ablation. The endpoint was not intended to capture all recurrent AF. It may miss recurrent AF managed conservatively, recurrent AF accepted as permanent, recurrence treated only with antiarrhythmic drug therapy or outpatient follow‐up, asymptomatic or low‐burden recurrence, and recurrence documented or treated outside the study center. All patients underwent standardized 7‐day Holter monitoring approximately 90 days after ablation followed by clinical review; thereafter, long‐term registry ascertainment was based on verifiable same‐center repeat PVI or electrical cardioversion. The nested active 2022 cohort was therefore analyzed separately because it had broader recurrence ascertainment. Patients were actively followed through the end of 2024. Patients without complete recurrence information were contacted directly between September and December 2024 and asked about documented atrial arrhythmia recurrence and recurrence‐related symptoms. When recurrence or compatible symptoms were reported, the recurrence date was recorded based on patient report and available clinical documentation. Patients who reported no recurrence or recurrence‐related symptoms underwent additional 72‐h Holter monitoring, which was reviewed as part of the active follow‐up process. Active follow‐up ascertainment was completed in early 2025.

Registry sensitivity analyses applied a 90‐day blanking period and one‐year outcome windows among patients with at least 365 days of potential administrative observation. In the active 2022 cohort, recurrence was defined as any documented recurrent atrial arrhythmia, including AF, atrial flutter, or atrial tachycardia when documented, after the index ablation based on direct patient contact, chart review, available external records, electrocardiography/Holter documentation, cardioversion, repeat ablation, or documented recurrence‐related care. Patients without documented recurrence were censored at the last available follow‐up date. Active time‐to‐event analyses were restricted to patients with valid event or censoring dates.

### 2.7 Elderly Vulnerability Phenotype

A prespecified exploratory analysis assessed whether a subset of elderly patients had outcomes comparable to those of the 50–74 years reference group. Within patients aged ≥ 75 years, candidate variables were screened using univariable Cox models for signal detection only. Candidate variables included sex, AF phenotype, BMI categories, heart failure, hypertension, diabetes, coronary disease, chronic kidney disease, stroke/TIA, valve disease/surgery, obstructive sleep apnea, left ventricular ejection fraction, procedural variables, and available laboratory markers. False discovery rate q‐values were calculated for the candidate screen.

A simple elderly clinical vulnerability score was then constructed using four clinically interpretable baseline features: documented persistent AF, heart failure, chronic kidney disease, and valve disease or prior valve surgery. These variables were selected because they were clinically plausible markers of advanced atrial or systemic vulnerability and could be identified during routine clinical assessment without requiring specialized imaging, complex biomarker panels, or other demanding measurements. Persistent AF was included as a marker of more advanced atrial substrate; heart failure as a marker of hemodynamic and structural cardiac vulnerability; chronic kidney disease as a marker of systemic comorbidity associated with adverse cardiovascular remodeling; and valve disease or prior valve surgery as a marker of chronic atrial loading and structural substrate. The score was intentionally limited to these readily available variables to preserve clinical usability and interpretability.

One point was assigned for each feature, and the score was categorized as 0, 1, or ≥ 2. Score 0 defined the elderly low‐vulnerability phenotype: age ≥ 75 years with no documented persistent AF, no heart failure, no chronic kidney disease, and no valve disease or prior valve surgery. The score was exploratory, internally derived, and intended to support hypothesis generation rather than function as an externally validated risk prediction tool.

### 2.8 Subgroup and Extreme‐Age Analyses

Age‐group analyses were repeated within sex and AF‐phenotype strata. Global age‐group‐by‐sex and age‐group‐by‐AF‐phenotype interactions were tested in adjusted Cox models.

Extreme‐age analyses were exploratory. Ages < 30 years, 30–84 years, and ≥ 85 years were evaluated in both cohorts. Because those groups were small, a registry‐only sensitivity analysis using < 40 years, 40–80 years, and > 80 years was also summarized to provide a more stable description of very young and very old patients.

### 2.9 Statistical Analysis

Continuous variables are summarized as median [interquartile range] unless otherwise specified, and categorical variables as *n*/*N* (%). Group comparisons used the Kruskal–Wallis test for continuous variables and the chi‐square or Fisher exact test for categorical variables, as appropriate. Laboratory‐panel *p*‐values were corrected with the Benjamini‐Hochberg false‐discovery‐rate method. Kaplan–Meier curves were compared using the log‐rank test and displayed through 30 months, with number‐at‐risk tables. Cox proportional hazards models estimated hazard ratios (HRs) with 95% confidence intervals (CIs). Logistic regression was used as a complementary binary‐endpoint analysis.

The main registry‐adjusted model included age group, sex, BMI per 5 kg/m^2^, persistent AF, hypertension, diabetes mellitus, heart failure, coronary artery disease/PCI/CABG, chronic kidney disease, cryoballoon PVI, and concomitant CTI/atrial flutter ablation. To address whether the composite registry endpoint was driven by repeat ablation or cardioversion, we performed separate component analyses for repeat PVI alone and electrical cardioversion alone. Cox models for these component endpoints used the same covariate structure as the main adjusted registry model. Component analyses were also repeated after applying the 90‐day blanking period. The active adjusted model included age group, sex, persistent AF, hypertension, diabetes mellitus, heart failure, coronary artery disease/PCI/CABG, and chronic kidney disease; a BMI‐adjusted active sensitivity model used BMI mapped from the registry when available. Missing data were not imputed; analyses used available cases. Two‐sided *p* < 0.05 was considered statistically significant. All analyses used only observed data; no synthetic observations or simulated outcomes were generated.

## 3 Results

### 3.1 Cohort Architecture and Age‐Group Selection

The registry cohort comprised 2559 first‐eligible PVI patients, with 580 same‐center repeat PVI/cardioversion events. The active 2022 cohort comprised 666 PVI patients; 343 had known active recurrence status, and 319 contributed valid active time‐to‐event data with 165 timed recurrence events. Active follow‐up architecture by age group, including the number with known recurrence status, valid time‐to‐event data, and timed recurrence events, is shown in Table S1. Candidate age cutoffs showed that < 40 years was clinically attractive but sparse. The selected categories of < 50, 50–74, and ≥ 75 years produced significant Kaplan–Meier separation in both cohorts and retained interpretable event counts.

### 3.2 Baseline Phenotype by Age Group

Older patients had a more comorbid clinical phenotype, whereas the < 50 years group represented a smaller, more male‐predominant early‐onset ablation population (Table 1). In the registry cohort, patients aged ≥ 75 years had higher rates of female sex than those aged 50–74 years and < 50 years, 49.6% versus 32.7% versus 14.2%, *p* < 0.001, persistent AF, 48.8% versus 39.8% versus 19.7%, *p* < 0.001, heart failure, 9.6% versus 6.7% versus 2.4%, *p* = 0.005, hypertension, 65.4% versus 56.8% versus 17.3%, *p* < 0.001, stroke/TIA, 9.1% versus 6.1% versus 3.9%, *p* = 0.015, coronary artery disease, 40.6% versus 24.0% versus 4.7%, *p* < 0.001, chronic kidney disease, 32.2% versus 11.3% versus 0.8%, *p* < 0.001, and valve disease/surgery, 40.8% versus 26.6% versus 19.7%, *p* < 0.001. The valve disease/surgery component was heterogeneous and included mitral insufficiency, tricuspid insufficiency, aortic insufficiency, aortic stenosis, and prior valve surgery. Younger patients had higher BMI, 26.0 kg/m^2^ [24.0–31.0] in those aged < 50 years versus 27.0 kg/m^2^ [24.0–30.0] in those aged 50–74 years and 25.0 kg/m^2^ [23.0–28.0] in those aged ≥ 75 years, *p* < 0.001, but substantially lower vascular and renal comorbidity. Within the ≥ 75‐year registry group, median age was 79 years [76–82]. Of 625 patients, 408 (65.3%) were aged 75–80 years, 176 (28.2%) were aged 81–84 years, and 41 (6.6%) were aged ≥ 85 years (Table S2).

**TABLE 1:** Baseline characteristics in the principal registry cohort by age group.

Characteristic	< 50 years	50–74 years	≥ 75 years	*p*
Patients, *n*	127	1807	625	
Age, years	44.0 [40.0, 47.0]	65.0 [59.0, 70.0]	79.0 [76.0, 82.0]	< 0.001
BMI, kg/m^2^	26.0 [24.0, 31.0]	27.0 [24.0, 30.0]	25.0 [23.0, 28.0]	< 0.001
Female sex	18/127 (14.2%)	591/1807 (32.7%)	310/625 (49.6%)	< 0.001
Persistent AF	25/127 (19.7%)	719/1807 (39.8%)	305/625 (48.8%)	< 0.001
Heart failure	3/127 (2.4%)	121/1807 (6.7%)	60/625 (9.6%)	0.005
Hypertension	22/127 (17.3%)	1027/1807 (56.8%)	409/625 (65.4%)	< 0.001
Diabetes mellitus	3/127 (2.4%)	198/1807 (11.0%)	73/625 (11.7%)	0.007
Stroke/TIA	5/127 (3.9%)	110/1807 (6.1%)	57/625 (9.1%)	0.015
Coronary artery disease	6/127 (4.7%)	433/1807 (24.0%)	254/625 (40.6%)	< 0.001
Chronic kidney disease	1/127 (0.8%)	205/1807 (11.3%)	201/625 (32.2%)	< 0.001
Valve disease/surgery	25/127 (19.7%)	481/1807 (26.6%)	255/625 (40.8%)	< 0.001
Administrative follow‐up, months	23.9 [13.9, 34.4]	21.9 [11.0, 34.9]	18.6 [9.4, 30.6]	< 0.001

*Note:* Values are median [interquartile range] or *n*/*N* (%).

Abbreviations: AF, atrial fibrillation; BMI, body mass index; PVI, pulmonary vein isolation; RF, radiofrequency; TIA, transient ischemic attack.

### 3.3 Registry Endpoint Components

The primary registry composite endpoint increased with age, but the component analysis showed different patterns for repeat ablation and cardioversion (Table 2). Repeat PVI rates were similar across age groups, whereas electrical cardioversion increased markedly with age. In adjusted Cox models, age ≥ 75 years was not associated with repeat PVI alone, HR: 0.95, 95% CI: 0.73–1.24, *p* = 0.705, but was associated with electrical cardioversion alone, HR: 1.63, 95% CI: 1.30–2.04, *p* < 0.001. The same pattern persisted after a 90‐day blanking period. Selected procedural and follow‐up characteristics by age group are shown in Table S4.

**TABLE 2:** Registry endpoint components and adjusted Cox models by age group.

Endpoint	< 50 years	50–74 years	> = 75 years	Adjusted HR: < 50 versus 50–74	Adjusted HR: > = 75 versus 50–74
Composite: repeat PVI or cardioversion	18/127 (14.2%)	376/1807 (20.8%)	186/625 (29.8%)	0.86 (0.53–1.39); *p* = 0.534	1.35 (1.12–1.64); *p* = 0.002
Repeat PVI alone	16/127 (12.6%)	251/1807 (13.9%)	88/625 (14.1%)	1.10 (0.65–1.84); *p* = 0.729	0.95 (0.73–1.24); *p* = 0.705
Electrical cardioversion alone	5/127 (3.9%)	236/1807 (13.1%)	149/625 (23.8%)	0.43 (0.18–1.05); *p* = 0.064	1.63 (1.30–2.04); *p* < 0.001
Composite after 90‐day blanking	17/127 (13.4%)	333/1807 (18.4%)	164/625 (26.2%)	0.91 (0.55–1.50); *p* = 0.708	1.37 (1.12–1.69); *p* = 0.002
Repeat PVI alone after 90‐day blanking	16/127 (12.6%)	248/1807 (13.7%)	86/625 (13.8%)	1.12 (0.66–1.88); *p* = 0.675	0.94 (0.72–1.23); *p* = 0.660
Electrical cardioversion alone after 90‐day blanking	4/127 (3.1%)	178/1807 (9.9%)	120/625 (19.2%)	0.46 (0.17–1.26); *p* = 0.131	1.79 (1.39–2.31); *p* < 0.001

### 3.4 Medication and Laboratory Phenotype

Medication patterns increased with the comorbidity burden with age (Table S3). Older patients were more often prescribed beta‐blockers, 62.5% versus 59.4% versus 49.6%, *p* = 0.026, ACE inhibitor/ARB/ARNI therapy, 42.1% versus 35.9% versus 11.4%, *p* < 0.001, Class III antiarrhythmics, 17.3% versus 12.1% versus 3.3%, *p* < 0.001, and diuretics, 29.6% versus 17.2% versus 3.1%, *p* < 0.001. Class I antiarrhythmic use was higher in younger patients, 27.6% versus 17.1% versus 8.6%, *p* < 0.001. Older patients had lower hemoglobin (13.8 vs. 14.5 vs. 15.3 g/dL), lower platelets (215 vs. 232 vs. 236), and lower eGFR (63.0 vs. 78.0 vs. 90.0), all *q* < 0.001, with higher creatinine (1.00 vs. 0.95 vs. 0.90), *q* < 0.001. LDL cholesterol decreased with age, 96 versus 114 versus 122, *q* < 0.001.

### 3.5 Primary Age‐Group Outcome Analyses

**FIGURE 1:**
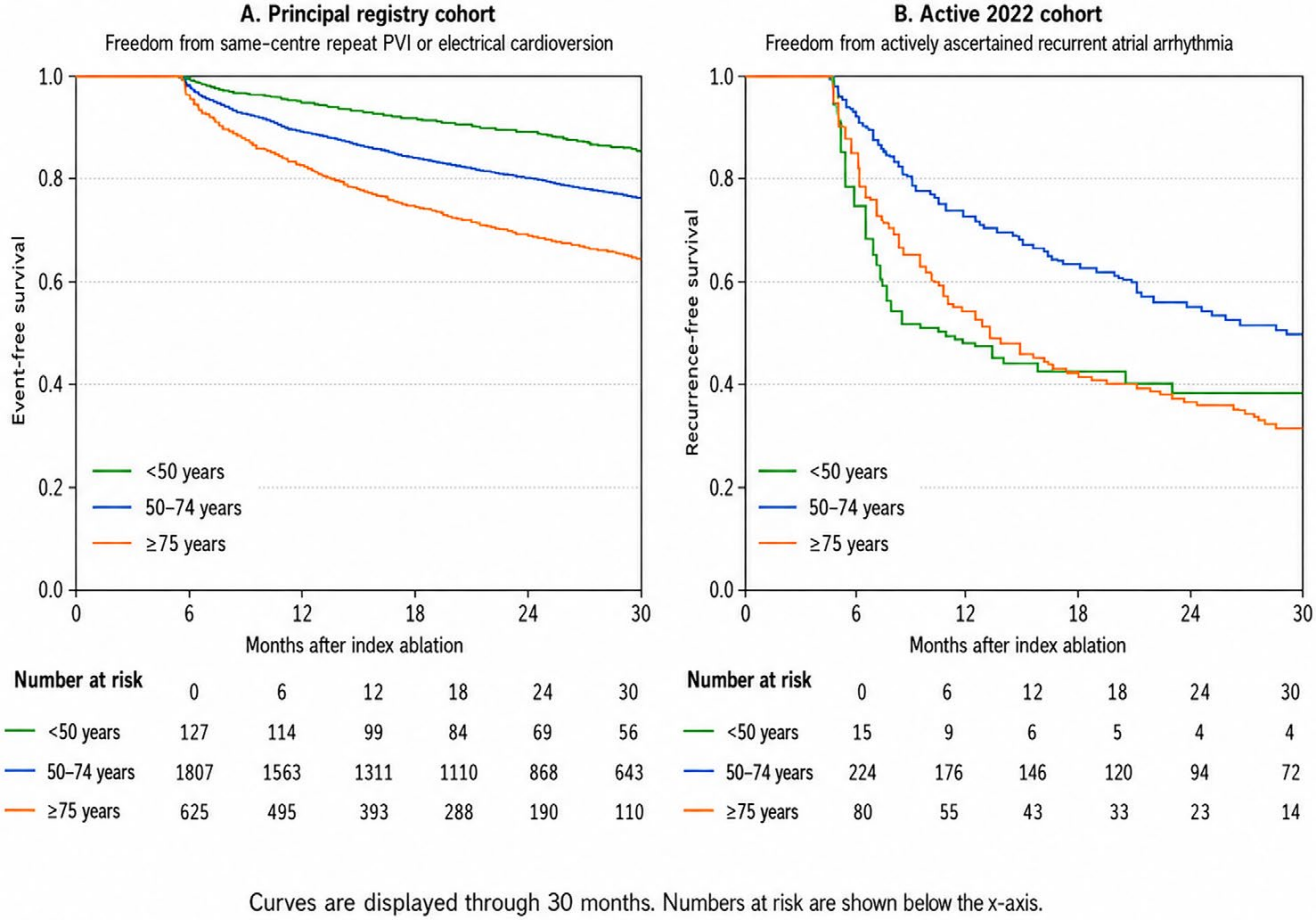
Kaplan–Meier freedom from recurrence‐related outcomes by age group. Left panel, principal registry cohort: Freedom from same‐center repeat PVI or electrical cardioversion. Right panel, nested active 2022 cohort: Freedom from actively ascertained recurrent atrial arrhythmia in patients with valid active event or censoring dates. Curves are displayed through 30 months. Numbers at risk are shown below the *x*‐axis. Events within 90 days were excluded, so curves remain at 1.0 until the first qualifying post‐blanking event. Legend: < 50 years, 50–74 years, and ≥ 75 years. PVI, pulmonary vein isolation.

Kaplan–Meier analysis demonstrated significant separation of freedom from same‐center repeat PVI/cardioversion in the registry cohort (log‐rank *p* < 0.001) and freedom from actively ascertained recurrence in the active cohort (log‐rank *p* = 0.037; Figure 1). In the registry cohort, same‐center repeat PVI/cardioversion increased from 14.2% in patients aged < 50 years to 20.8% in those aged 50–74 years and 29.8% in those aged ≥ 75 years.

Crude event percentages were higher in the active cohort than in the registry because the two analyses used different endpoints and ascertainment. Among patients with valid active time‐to‐event data, recurrence occurred in 105/224 (46.9%) patients aged 50–74 years and 51/80 (63.8%) patients aged ≥ 75 years, compared with registry same‐center intervention rates of 20.8% and 29.8%, respectively. The registry required a verified same‐center repeat PVI or electrical cardioversion, whereas active follow‐up captured any documented recurrent atrial arrhythmia through direct patient contact, clinical and available external records, electrocardiography/Holter documentation, cardioversion, repeat ablation, or other documented recurrence‐related care. These percentages therefore represent different ascertainment frameworks and should not be interpreted as directly comparable estimates of total recurrence.

In the registry‐adjusted Cox model, the overall age‐group effect was significant and was driven by the ≥ 75 years group. Compared with age 50–74 years, age ≥ 75 years was associated with a higher hazard of same‐center recurrence‐related intervention (HR: 1.35, 95% CI: 1.12–1.64, *p* = 0.002), and this persisted after a 90‐day blanking period (HR: 1.37, 95% CI: 1.12–1.69, *p* = 0.002; Table 3). Age < 50 years was not independently associated with a higher hazard compared with age 50–74 years. The active 2022 cohort showed a similar elderly signal using actively ascertained recurrence (adjusted HR: 1.46, 95% CI: 1.01–2.11, *p* = 0.042), although the < 50 year active time‐to‐event group was small and imprecise.

**TABLE 3:** Main adjusted outcome models by age group.

Cohort/model	Age < 50 versus 50–74	*p*	Age ≥ 75 versus 50–74	*p*
Registry adjusted logistic	OR 0.87 (0.51–1.48)	0.618	OR 1.43 (1.14–1.80)	0.002
Registry unadjusted Cox	HR 0.65 (0.40–1.04)	0.072	HR 1.55 (1.30–1.85)	< 0.001
Registry adjusted Cox	HR 0.86 (0.53–1.39)	0.534	HR 1.35 (1.12–1.64)	0.002
Registry adjusted Cox after 90‐day blanking	HR 0.91 (0.55–1.50)	0.708	HR 1.37 (1.12–1.69)	0.002
Active adjusted logistic	OR 2.34 (0.81–6.82)	0.118	OR 1.88 (1.07–3.29)	0.028
Active unadjusted Cox	HR 1.44 (0.73–2.86)	0.293	HR 1.52 (1.09–2.13)	0.014
Active adjusted Cox	HR 1.66 (0.82–3.36)	0.159	HR 1.46 (1.01–2.11)	0.042
Active BMI‐adjusted Cox sensitivity	HR 1.80 (0.88–3.65)	0.105	HR 1.58 (1.08–2.30)	0.017

*Note:* Reference group is age 50–74 years. Registry adjusted models included age group, sex, BMI, persistent AF, hypertension, diabetes mellitus, heart failure, CAD/PCI/CABG, CKD, cryoballoon PVI identified from procedure reports, and concomitant CTI/atrial flutter ablation identified from procedure reports. Active models used available clinical covariates; BMI was added in sensitivity analysis.

### 3.6 Subgroup Analyses by Sex and AF Phenotype

The elderly signal remained clinically visible in sex‐stratified and AF‐phenotype‐stratified analyses (Table S5 and Figures S1 and S2). In the registry cohort, age ≥ 75 years was directionally associated with higher risk in both men, adjusted HR: 1.29, 95% CI: 0.99–1.68, *p* = 0.057, and women, adjusted HR: 1.43, 95% CI: 1.07–1.90, *p* = 0.015. However, formal interaction testing did not support robust effect modification by sex, global interaction *p* = 0.452 in the registry cohort and *p* = 0.343 in the active cohort (Table S6). In AF‐phenotype analyses, the strongest registry signal was observed among patients with documented paroxysmal AF, adjusted HR: 1.92, 95% CI: 1.20–3.07, *p* = 0.007, compared with persistent AF, adjusted HR: 1.26, 95% CI: 0.98–1.62, *p* = 0.076. The global age‐by‐AF‐phenotype interaction was not significant, *p* = 0.121 in the registry cohort and *p* = 0.587 in the active cohort. These analyses are therefore best interpreted as clinically informative rather than definitive evidence that the age effect differs by sex or AF phenotype.

### 3.7 Elderly Low‐Vulnerability Phenotype

The excess risk among elderly patients was not uniform (Table 4). The four‐item elderly clinical vulnerability score separated older patients by recurrence‐related event burden in both cohorts. Elderly Score 0 patients were not significantly different from the age 50–74 reference group in registry analysis, HR: 1.13, 95% CI: 0.79–1.63, *p* = 0.500, or active analysis, HR: 1.01, 95% CI: 0.49–2.09, *p* = 0.975. By contrast, elderly patients with Score ≥ 2 had higher event burden in the registry cohort, HR: 1.85, 95% CI: 1.45–2.35, *p* < 0.001, and in the active 2022 cohort, HR: 1.95, 95% CI: 1.23–3.12, *p* = 0.005.

**TABLE 4:** Elderly clinical vulnerability score groups versus the 50–74 years reference group.

Group	Registry events/patients	Registry event rate	Registry 24‐month event‐free survival	Registry adjusted HR versus 50–74	Active TTE events/patients	Active event rate	Active 24‐month event‐free survival	Active adjusted HR versus 50–74
Age 50–74 reference	376/1807	20.8%	79.7%	Reference	105/224	46.9%	55.5%	Reference
Age ≥ 75, Score 0	36/147	24.5%	75.6%	1.13 (0.79–1.63), *p* = 0.500	8/17	47.1%	52.9%	1.01 (0.49–2.09), *p* = 0.975
Age ≥ 75, Score 1	58/219	26.5%	76.8%	1.19 (0.89–1.60), *p* = 0.244	19/31	61.3%	41.9%	1.32 (0.80–2.18), *p* = 0.271
Age ≥ 75, Score ≥ 2	92/259	35.5%	63.7%	1.85 (1.45–2.35), *p* < 0.001	24/32	75.0%	30.9%	1.95 (1.23–3.12), *p* = 0.005

*Note:* Score components were documented persistent AF, heart failure, CKD defined as KDIGO G3a or worse, and valve disease/surgery. One point was assigned for each component. Score 0 defined the low‐vulnerability elderly phenotype. Registry and active adjusted models used the age 50–74 group as the reference and did not adjust for the score components themselves.

Abbreviation: TTE, time‐to‐event.

### 3.8 Exploratory Analyses of Very Young and Very Old

The most extreme age strata were rare (Table S7 and Figure S3). In the primary extreme‐age registry analysis, only six patients were aged < 30 years and 41 were aged ≥ 85 years, with crude endpoint rates of 33.3% and 29.3%, respectively, compared with 22.5% in those aged 30–84 years (log‐rank *p* = 0.395). Adjusted estimates were imprecise and non‐significant for both < 30 years, HR: 1.67, 95% CI: 0.41–6.78, *p* = 0.471, and ≥ 85 years, HR: 1.24, 95% CI: 0.68–2.28, *p* = 0.480. The active cohort contained only three patients aged < 30 years and four aged ≥ 85 years, so active extreme‐age Cox modeling was not reliable. A broader registry‐only sensitivity analysis using < 40, 40–80, and > 80 years showed higher crude endpoint rates in very old patients, 30.4% versus 22.0% versus 13.8%, log‐rank *p* = 0.002, but the adjusted > 80 years signal attenuated after 90‐day blanking, HR: 1.12, 95% CI: 0.82–1.52, *p* = 0.492.

### 3.9 Procedural Safety in the Active 2022 Cohort

Age‐stratified procedural safety in the active 2022 cohort is shown in Table 5. Any documented procedural complication occurred in 32/666 patients (4.8%), with rates of 3.1%, 4.0%, and 7.6% in the < 50, 50–74, and ≥ 75 years groups, respectively. Vascular complications occurred in 19/666 patients (2.9%), pericardial effusion/tamponade in 3/666 (0.5%), procedural stroke/TIA in 1/666 (0.2%), and CPR in 1/666 (0.2%). Major bleeding/transfusion surrogate events occurred in 5/666 patients (0.8%), and no procedure‐related deaths occurred.

**TABLE 5:** Procedural complications in the active 2022 cohort by age group.

Complication category	< 50 years	50–74 years	> = 75 years	Total	*p*
AV fistula	0/32 (0.0%)	8/476 (1.7%)	3/158 (1.9%)	11/666 (1.7%)	0.741
Pseudoaneurysm/aneurysm	0/32 (0.0%)	3/476 (0.6%)	3/158 (1.9%)	6/666 (0.9%)	0.295
Groin operation	0/32 (0.0%)	3/476 (0.6%)	1/158 (0.6%)	4/666 (0.6%)	0.903
Major bleeding/transfusion	0/32 (0.0%)	3/476 (0.6%)	2/158 (1.3%)	5/666 (0.8%)	0.638
Transfusion	0/32 (0.0%)	0/476 (0.0%)	2/158 (1.3%)	2/666 (0.3%)	0.040
Air embolism	0/32 (0.0%)	0/476 (0.0%)	0/158 (0.0%)	0/666 (0.0%)	Not tested
Phrenic nerve lesion	1/32 (3.1%)	1/476 (0.2%)	2/158 (1.3%)	4/666 (0.6%)	0.055
CPR	0/32 (0.0%)	0/476 (0.0%)	1/158 (0.6%)	1/666 (0.2%)	0.200
TIA/Stroke	0/32 (0.0%)	1/476 (0.2%)	0/158 (0.0%)	1/666 (0.2%)	1.000
Pericardial effusion/tamponade	0/32 (0.0%)	1/476 (0.2%)	2/158 (1.3%)	3/666 (0.5%)	0.271
Procedure‐related death	0/32 (0.0%)	0/476 (0.0%)	0/158 (0.0%)	0/666 (0.0%)	Not tested
Any documented procedural complication	1/32 (3.1%)	19/476 (4.0%)	12/158 (7.6%)	32/666 (4.8%)	0.167
Vascular complication	0/32 (0.0%)	12/476 (2.5%)	7/158 (4.4%)	19/666 (2.9%)	0.280

*Note: p*‐values are descriptive; exact tests were used for sparse complication endpoints. TIA: transient ischemic attack.

## 4 Discussion

In this single‐center study of patients undergoing AF ablation with PVI, age ≥ 75 years was associated with a higher burden of same‐center recurrence‐related rhythm intervention in the registry cohort and higher actively ascertained recurrence in the active 2022 cohort. However, the main finding is not that older age alone predicts poorer outcomes. Rather, the burden of recurrence‐related events among older patients was heterogeneous. Elderly patients without documented persistent AF, heart failure, chronic kidney disease, or valve disease/surgery did not have a statistically significant increase in registry recurrence‐related rhythm intervention hazard compared with those aged 50–74 years, whereas those with two or more vulnerability features had substantially higher recurrence‐related event burden.

These findings are consistent with contemporary evidence showing that AF ablation remains feasible in selected older patients, but recurrence risk is generally higher than in younger populations [6]. Boehmer et al. reported higher recurrence after cryoballoon PVI in patients aged ≥ 75 years, particularly among elderly patients with persistent AF [7]. Their systematic review also concluded that AF ablation in patients aged ≥ 75 years carries a higher risk of recurrence and procedure‐related complications than in younger patients. Metzner et al. similarly found favorable long‐term outcomes in selected patients aged ≥ 75 years with paroxysmal AF, but less favorable outcomes in those with persistent or long‐standing AF [8].

The age distribution within our elderly group is important when interpreting these findings. The ≥ 75‐year registry group had a median age of 79 years [76–82], and only 41/625 patients (6.6%) were aged ≥ 85 years, so the principal ≥ 75 years estimate largely reflects patients in their late 70s and early 80s rather than the very elderly. In the large prospective Japan Ablation Registry, which analyzed age in 5‐year strata through ≥ 85 years, arrhythmia recurrence did not differ significantly across age groups, whereas procedure‐related complications increased with age [9]. That external pattern is not identical to our actively ascertained recurrence signal, but it is compatible with our registry component analysis, in which repeat PVI did not increase with age and the composite age association was driven by cardioversion. Together, these observations support caution when extrapolating our main ≥ 75 years estimate to patients aged ≥ 85 years.

Our study extends this literature in three ways. First, it separates chronological age from a simple clinical vulnerability phenotype. Prior studies have generally compared age‐defined groups or examined age strata without incorporating a simple vulnerability phenotype [6, 7, 8, 9]. Our findings suggest that the excess risk in elderly patients is concentrated in those with markers of advanced atrial and systemic disease, including persistent AF, heart failure, chronic kidney disease, and valve disease/surgery. Second, we used two endpoint frameworks: a highly specific, same‐center, recurrence‐related intervention endpoint and a broader, actively ascertained recurrence endpoint. The consistency of the elderly signal across both frameworks supports the robustness of the association. Third, we show that elderly patients without these vulnerability features had outcomes similar to the 50–74 years reference group, arguing against using age ≥ 75 years as a stand‐alone reason to avoid PVI [10].

The registry endpoint should be interpreted as recurrence‐related care rather than continuous rhythm surveillance. Same‐center repeat PVI and electrical cardioversion are clinically meaningful events, but they are influenced by symptoms, referral patterns, patient preference, frailty, clinician judgment, and local rhythm‐control practice. Electrical cardioversion was counted as a recurrence‐related intervention in all age groups because it represents clinically recognized recurrent atrial arrhythmia requiring rhythm‐control treatment after PVI. However, the component analysis showed that the age‐related increase in the composite registry endpoint was driven predominantly by electrical cardioversion rather than repeat PVI. This pattern may partly reflect age‐related treatment selection after recurrence. Patients are older when recurrence occurs than at the index ablation, and advancing age, frailty, comorbidity, or clinician and patient preference may reduce the likelihood of redo ablation while favoring electrical cardioversion as a less invasive rhythm‐control intervention. The higher cardioversion rate in older patients should therefore not be interpreted as evidence of greater biological recurrence alone. The active 2022 cohort partly mitigated this limitation by including broader recurrence ascertainment, and the persistence of the elderly signal in that cohort supports the clinical relevance of the finding.

The vulnerability score provides a practical counseling framework, but it should not be interpreted as a validated risk‐prediction instrument. Persistent AF likely reflects a more advanced atrial substrate and a lower probability of durable rhythm control after PVI alone. Heart failure and chronic kidney disease may reflect systemic vulnerability, hemodynamic stress, inflammation, and altered atrial remodeling. Valve disease or previous valve surgery may indicate chronic atrial loading and structural substrate, although individual valve lesions may have different implications [11]. The valve‐disease component was heterogeneous and included mitral insufficiency, tricuspid insufficiency, aortic insufficiency, aortic stenosis, and prior valve surgery. Future studies should evaluate whether left‐sided moderate or severe valve disease carries stronger prognostic weight than milder or right‐sided lesions. These features are easily identifiable during routine clinical assessment. Their absence identified a low‐vulnerability elderly phenotype without a statistically significant excess registry hazard compared with the 50–74 year reference group, while their accumulation, particularly two or more features, identified a subgroup with markedly higher recurrence‐related rhythm‐intervention burden.

These findings have direct clinical implications. In older patients being considered for PVI ablation, counseling should avoid binary decisions based on chronological age. Patients aged ≥ 75 years with low clinical vulnerability may still have reasonable expectations for rhythm control. Conversely, those with multiple vulnerability features should receive more cautious counseling about recurrence risk, the potential need for cardioversion or repeat procedures, and the role of risk‐factor optimization, antiarrhythmic therapy, or rate‐control strategies [12]. This is particularly relevant for elderly patients with persistent AF and heart failure, a clinically challenging population that is overrepresented among vulnerable older patients. The present data do not support a categorical recommendation to avoid ablation in such patients, but they do support more cautious counseling. In elderly patients with persistent AF and heart failure, ablation may still be reasonable when symptoms, tachycardia‐mediated cardiomyopathy, recurrent decompensation, patient goals, and acceptable procedural risk favor rhythm control. Conversely, when multiple vulnerability features coexist, the expected need for cardioversion, repeat rhythm‐control interventions, or transition to antiarrhythmic‐drug or rate‐control strategies should be discussed explicitly. This approach aligns with contemporary guideline emphasis on shared decision‐making and individualized rhythm‐control selection [13] and is consistent with the REHEALTH AF Study, a prospective multicenter observational study in patients aged ≥ 80 years, in which ablation did not show a significant unadjusted or matched reduction in the primary composite outcome versus conservative management, but adjusted analyses suggested benefit and ablation was associated at 1 year with improved symptoms, quality of life, NT‐proBNP, left atrial diameter, and preservation of cognitive and frailty measures [14].

Subgroup analyses by sex and AF phenotype were clinically informative but not definitive. The elderly signal was directionally present in both men and women and appeared stronger in women in the registry cohort, but formal interaction testing did not support robust effect modification. Similarly, the strongest registry signal was observed among patients with documented paroxysmal AF, although the global age‐by‐AF‐phenotype interaction was not significant. This finding should not be interpreted as proof that age has a stronger effect on paroxysmal AF. It may reflect selection, symptom burden, cardioversion practice, or unmeasured atrial substrate in older patients labeled as paroxysmal.

The extreme‐age analyses should be interpreted cautiously. Patients aged < 30 years and ≥ 85 years were rare, and estimates were imprecise. Only 41 registry patients were aged ≥ 85 years. A broader registry‐only comparison across < 40, 40–80, and > 80 years suggested higher crude event rates in very old patients, but the adjusted association for > 80 years patients attenuated after 90‐day blanking. These results support descriptive reporting rather than firm inference in the oldest and youngest age strata and limit extrapolation of the main ≥ 75 years estimate to the very elderly.

Overall, this study supports a shift from age‐based selection toward vulnerability‐informed counseling. Age ≥ 75 years identifies a population with higher average same‐center recurrence‐related intervention after PVI, but it does not define a uniform risk group. A simple phenotype based on persistent AF, heart failure, chronic kidney disease, and valve disease/surgery may help identify older patients with substantially higher recurrence‐related event burden, particularly when two or more vulnerability features are present. This score should be viewed as exploratory and hypothesis‐generating, requiring prospective external validation with standardized rhythm monitoring, detailed assessment of atrial substrate, and patient‐centered outcomes.

## 5 Limitations

This study has several limitations. First, this was a single‐center observational study. Treatment selection, ablation modality, additional lesion sets, follow‐up intensity, and post‐recurrence management were not randomized and may have changed over time. Second, the age cutoffs and elderly vulnerability phenotype were data‐guided and internally derived. The vulnerability score should therefore be interpreted as exploratory and hypothesis‐generating, not as an externally validated risk‐prediction tool.

Third, the registry endpoint captured same‐center repeat PVI or electrical cardioversion and was not designed to measure total arrhythmia burden under continuous rhythm surveillance. Although electrical cardioversion was counted as a recurrence‐related intervention in all age groups, the component analysis showed that the age‐related increase in the composite endpoint was driven predominantly by cardioversion rather than repeat PVI. Therefore, the registry endpoint likely reflects both clinically recognized recurrence and age‐related differences in post‐recurrence management strategy. Because treatment choice occurs after recurrence, management bias is possible: older patients may be less likely to undergo redo ablation and more likely to receive cardioversion. The registry endpoint may also miss recurrence managed conservatively, recurrence treated only with antiarrhythmic‐drug escalation or outpatient follow‐up, asymptomatic or low‐burden recurrence, and recurrence documented or treated outside the study center.

Fourth, the active 2022 cohort was nested within the same institutional registry framework and should not be considered external validation. Although active follow‐up was more intensive and included direct patient contact and additional 72‐h Holter monitoring when needed, it was not continuous rhythm monitoring and did not include systematic implantable‐monitor surveillance. Known active recurrence status was available in 343/666 patients, and valid active time‐to‐event data were available in 319/666 patients; therefore, selection and information bias remain possible. The higher crude event percentages in the active cohort reflect its broader recurrence ascertainment and should not be compared directly with the registry intervention endpoint.

Fifth, some score components had definitional limitations. The valve disease/surgery component was heterogeneous and combined mitral insufficiency, tricuspid insufficiency, aortic insufficiency, aortic stenosis, and prior valve surgery; mitral stenosis was not available as a separate structured field.

Sixth, structured procedural safety data were available only in the active 2022 cohort, not across the full registry cohort. Safety analyses were therefore descriptive, based on sparse event counts. Seventh, medication and laboratory comparisons were descriptive and should not be interpreted as causal mediators. Finally, the extreme‐age analyses were underpowered and should be considered hypothesis‐generating only.

## 6 Conclusion

Age ≥ 75 years was associated with higher same‐center recurrence‐related rhythm intervention in the registry cohort and higher actively ascertained recurrence in the active cohort. However, elderly patients were not homogeneous. Those without documented persistent AF, heart failure, chronic kidney disease, or valve disease/surgery did not have a statistically significant increase in registry recurrence‐related rhythm‐intervention hazard compared with patients aged 50–74 years, while those with multiple vulnerability features had substantially higher risk. Chronological age should inform counseling, but clinical vulnerability may better identify older patients with substantially higher recurrence‐related event burden after PVI.

## Funding

G.A.N. is supported by British Heart Foundation Research Excellence Award (RE/24/130031), British Heart Foundation Programme Grant (RG/17/3/32774), Medical Research Council Biomedical Catalyst Developmental Pathway Funding Scheme (MR/S037306/1), and NIHR i4i grant (NIHR204553). Mustafa Zakkar is supported by the British Heart Foundation award (CH/12/1/29419) to the University of Leicester and Leicester NIHR Biomedical Research Center (NIHR203327). Yazan Mohsen is supported by the Laducq foundation (grant number 23CVD04).

## Ethics Statement

The study was reviewed and ethically approved by the institutional review board of the University of Witten/Herdecke (S‐192/2023).

## Conflicts of Interest

The authors declare no conflicts of interest.

## Supporting information


**Table S1:** Active 2022 follow‐up architecture and actively ascertained recurrence by age group.
**Table S2:** Age distribution within the registry cohort aged ≥ 75 years.
**Table S3:** Selected medication and laboratory variables by age group.
**Table S4:** Selected procedural and follow‐up characteristics by age group.
**Table S5:** Subgroup outcome models by sex and AF phenotype.
**Table S6:** Formal interaction testing.
**Table S7:** Extreme‐age exploratory analyses.
**Figure S1:** Registry subgroup Kaplan–Meier analyses. A, Principal registry cohort stratified by sex. B, Principal registry cohort stratified by AF phenotype.
**Figure S2:** Active 2022 subgroup Kaplan–Meier analyses. A, Active cohort stratified by sex. B, Active cohort stratified by AF phenotype.
**Figure S3:** Exploratory very young and very old analyses. A, Principal registry cohort Kaplan–Meier freedom from same‐center repeat PVI or electrical cardioversion for < 30, 30–84, and ≥ 85 years. B, Registry‐only sensitivity Kaplan–Meier freedom from same‐center repeat PVI or electrical cardioversion for < 40, 40–80, and > 80 years. C, Registry‐only sensitivity crude primary endpoint rates for < 40, 40–80, and > 80 years.

## Data Availability

The data that support the findings of this study are available from the corresponding author upon reasonable request.
