# Supplementary material for: Clinical Vulnerability Refines Rhythm‐Related Outcomes in Older Patients Undergoing Pulmonary Vein Isolation for Atrial Fibrillation: An Observational Cohort Study
Source: J Arrhythm. 2026 Sep 25;42(5):e70461. doi: 10.1002/joa3.70461 (PMC13615338; doi:10.1002/joa3.70461)
Supplement: Supplementary file 1 [file JOA3-42-e70461-s001.docx]

**Supplementary Material**

**Clinical Vulnerability Refines Rhythm-Related Outcomes in Older Patients Undergoing Pulmonary Vein Isolation for Atrial Fibrillation: An Observational Cohort Study**

This file contains supplementary tables and figures cited in the updated main manuscript. Numbering has been revised to match the in-text citations.

# Supplementary Tables

**Supplementary Table S1. Active 2022 follow-up architecture and actively ascertained recurrence by age group.**

| **Age group** | **Full active cohort, n** | **Known active recurrence status, n/N (%)** | **Valid active TTE data, n/N (%)** | **Timed recurrence events / valid TTE (%)** |
| --- | --- | --- | --- | --- |
| <50 years | 32 | 17/32 (53.1%) | 15/32 (46.9%) | 9/15 (60.0%) |
| 50-74 years | 476 | 239/476 (50.2%) | 224/476 (47.1%) | 105/224 (46.9%) |
| ≥75 years | 158 | 87/158 (55.1%) | 80/158 (50.6%) | 51/80 (63.8%) |
| Total | 666 | 343/666 (51.5%) | 319/666 (47.9%) | 165/319 (51.7%) |

Values describe the architecture of the nested active 2022 cohort. The active endpoint used broader recurrence ascertainment than the registry endpoint. Active recurrence could be identified through direct patient contact, clinical or available external records, electrocardiography/Holter documentation, cardioversion, repeat ablation, or other documented recurrence-related care. TTE = time-to-event.

**Supplementary Table S2. Age distribution within the registry cohort aged ≥75 years.**

| **Age stratum** | **Patients, n (%)** |
| --- | --- |
| 75-80 years | 408/625 (65.3%) |
| 81-84 years | 176/625 (28.2%) |
| ≥85 years | 41/625 (6.6%) |
| Total ≥75 years | 625 (100%) |

Median age in the ≥75-year registry group was 79 years [interquartile range 76-82]. The strata above are derived from the registry age distribution and the prespecified extreme-age sensitivity analyses.

**Supplementary Table S3. Selected medication and laboratory variables by age group.**

| **Variable** | **<50 years** | **50-74 years** | **≥75 years** | **p value / FDR** |
| --- | --- | --- | --- | --- |
| Beta-blocker | 61/123 (49.6%) | 1032/1738 (59.4%) | 379/606 (62.5%) | 0.026 |
| ACE inhibitor/ARB/ARNI | 14/123 (11.4%) | 624/1738 (35.9%) | 255/606 (42.1%) | <0.001 |
| Any oral anticoagulation | 96/127 (75.6%) | 1293/1807 (71.6%) | 445/625 (71.2%) | 0.594 |
| Class I antiarrhythmic | 34/123 (27.6%) | 298/1738 (17.1%) | 52/606 (8.6%) | <0.001 |
| Class III antiarrhythmic | 4/123 (3.3%) | 210/1738 (12.1%) | 105/606 (17.3%) | <0.001 |
| Any diuretic | 4/127 (3.1%) | 310/1807 (17.2%) | 185/625 (29.6%) | <0.001 |
| Hemoglobin, g/dL | 15.3 [14.3, 15.6] | 14.5 [13.7, 15.4] | 13.8 [12.9, 14.8] | q<0.001 |
| Platelets | 236 [206, 277] | 232 [199, 268] | 215 [183, 254] | q<0.001 |
| Creatinine | 0.90 [0.80, 1.02] | 0.95 [0.83, 1.09] | 1.00 [0.86, 1.20] | q<0.001 |
| eGFR CKD-EPI | 90.0 [88.8, 90.0] | 78.0 [66.0, 89.0] | 63.0 [50.0, 75.0] | q<0.001 |
| HDL cholesterol | 48.0 [42.8, 56.0] | 54.0 [45.0, 66.0] | 61.0 [51.0, 72.2] | q<0.001 |
| LDL cholesterol | 122 [96, 143] | 114 [84, 142] | 96 [72, 129] | q<0.001 |
| Glucose | 91.0 [83.0, 99.0] | 95.0 [87.0, 106.0] | 97.0 [90.0, 109.0] | q<0.001 |

Values are n/N (%) or median [interquartile range]. Laboratory p values were corrected across the laboratory panel using the Benjamini-Hochberg false-discovery-rate method. ACE = angiotensin-converting enzyme; ARB = angiotensin receptor blocker; ARNI = angiotensin receptor neprilysin inhibitor; CKD-EPI = Chronic Kidney Disease Epidemiology Collaboration; eGFR = estimated glomerular filtration rate; FDR = false discovery rate; HDL = high-density lipoprotein; LDL = low-density lipoprotein.

**Supplementary Table S4. Selected procedural and follow-up characteristics by age group.**

| **Characteristic** | **<50 years** | **50-74 years** | **≥75 years** | **p value** |
| --- | --- | --- | --- | --- |
| CTI/atrial flutter ablation | 9/127 (7.1%) | 188/1807 (10.4%) | 70/625 (11.2%) | 0.383 |
| Substrate-guided ablation | 22/127 (17.3%) | 416/1807 (23.0%) | 175/625 (28.0%) | 0.008 |
| Administrative follow-up, months | 23.9 [13.9, 34.4] | 21.9 [11.0, 34.9] | 18.6 [9.4, 30.6] | <0.001 |

Values are n/N (%) or median [interquartile range]. CTI = cavotricuspid isthmus; PVI = pulmonary vein isolation.

**Supplementary Table S5. Subgroup outcome models by sex and AF phenotype.**

| **Cohort** | **Subgroup** | **Events by age group** | **Age ≥75 vs 50-74 adjusted HR** | **p value** |
| --- | --- | --- | --- | --- |
| Registry | Men | <50: 17/109; 50-74: 243/1216; ≥75: 87/315 | 1.29 (0.99-1.68) | 0.057 |
| Registry | Women | <50: 1/18; 50-74: 133/591; ≥75: 99/310 | 1.43 (1.07-1.90) | 0.015 |
| Active 2022 | Men | <50: 9/14; 50-74: 74/156; ≥75: 21/41 | 1.29 (0.74-2.25) | 0.370 |
| Active 2022 | Women | <50: 2/3; 50-74: 46/83; ≥75: 37/46 | 1.44 (0.85-2.46) | 0.175 |
| Registry | Paroxysmal AF | <50: 8/34; 50-74: 68/383; ≥75: 34/106 | 1.92 (1.20-3.07) | 0.007 |
| Registry | Persistent AF | <50: 4/25; 50-74: 208/719; ≥75: 111/305 | 1.26 (0.98-1.62) | 0.076 |
| Active 2022 | Paroxysmal AF | <50: 10/15; 50-74: 79/164; ≥75: 30/50 | 1.33 (0.82-2.18) | 0.253 |
| Active 2022 | Persistent AF | <50: 1/2; 50-74: 41/75; ≥75: 28/37 | 1.59 (0.91-2.79) | 0.104 |

Events by age group are crude recurrence or recurrence-related intervention counts within each subgroup. The table emphasises the age ≥75 years contrast. Estimates for age <50 years were imprecise after stratification. AF = atrial fibrillation; HR = hazard ratio.

**Supplementary Table S6. Formal interaction testing.**

| **Cohort** | **Modifier** | **Model N / events** | **Global age group × modifier p value** |
| --- | --- | --- | --- |
| Registry | Sex | 2413 / 547 | 0.452 |
| Active 2022 | Sex | 310 / 159 | 0.343 |
| Registry | AF phenotype | 1468 / 407 | 0.121 |
| Active 2022 | AF phenotype | 310 / 159 | 0.587 |

Interaction models did not show statistically robust evidence that the age effect differed by sex or AF phenotype. AF = atrial fibrillation.

**Supplementary Table S7. Extreme-age exploratory analyses.**

| **Analysis** | **Group sizes** | **Endpoint rates** | **Log-rank p** | **Adjusted Cox estimate** |
| --- | --- | --- | --- | --- |
| Primary extreme registry: <30 / 30-84 / ≥85 | 6 / 2512 / 41 | 33.3% / 22.5% / 29.3% | 0.395 | <30 vs 30-84 HR 1.67 (0.41-6.78), p=0.471; ≥85 vs 30-84 HR 1.24 (0.68-2.28), p=0.480 |
| Primary extreme active 2022: <30 / 30-84 / ≥85 | 3 / 659 / 4 | Known status too sparse | Not stable | Primary active Cox not reliable because only 1 <30 and 2 ≥85 patients had usable TTE data |
| Registry-only sensitivity: <40 / 40-80 / >80 | 29 / 2313 / 217 | 13.8% / 22.0% / 30.4% | 0.002 | <40 vs 40-80 HR 0.85 (0.32-2.29), p=0.749; >80 vs 40-80 HR 1.32 (1.00-1.74), p=0.050 |
| Registry-only sensitivity after 90-day blanking | 29 / 2313 / 217 | 10.3% / 19.8% / 24.0% | 0.079 | >80 vs 40-80 HR 1.12 (0.82-1.52), p=0.492 |

Extreme-age results are exploratory and should not replace the main <50, 50-74, and ≥75 years analysis. HR = hazard ratio; TTE = time-to-event.

# Supplementary Figures

Supplementary Figure S1. Registry subgroup Kaplan-Meier analyses. A, Principal registry cohort stratified by sex. B, Principal registry cohort stratified by AF phenotype.

**Supplementary Figure S1A**

**Supplementary Figure S1B**

Supplementary Figure S2. Active 2022 subgroup Kaplan-Meier analyses. A, Active cohort stratified by sex. B, Active cohort stratified by AF phenotype.

**Supplementary Figure S2A**

**Supplementary Figure S2B**

Supplementary Figure S3. Exploratory very young and very old analyses. A, Principal registry cohort Kaplan-Meier freedom from same-centre repeat PVI or electrical cardioversion for <30, 30-84, and ≥85 years. B, Registry-only sensitivity Kaplan-Meier freedom from same-centre repeat PVI or electrical cardioversion for <40, 40-80, and >80 years. C, Registry-only sensitivity crude primary endpoint rates for <40, 40-80, and >80 years.

**Supplementary Figure S3A**

**Supplementary Figure S3B**

**Supplementary Figure S3C**
